# Physicochemical and Microstructural Characterization of Sunflower Husk Ash and Thermal Power Plant Electrofilter Ash: A Preliminary Assessment for Construction Applications

**DOI:** 10.3390/ma19194233

**Published:** 2026-10-06

**Authors:** Cosmin Butura, Robert Cătălin Ciocoiu, Doina Roxana Trușcă, Gabriel Dobri, Alexandra Banu

**Affiliations:** 1 Doctoral School, Faculty of Material Science and Engineering, National University of Science and Technology POLITEHNICA Bucharest, 060042 Bucharest, Romania; 2 Faculty of Material Science and Engineering, National University of Science and Technology POLITEHNICA Bucharest, 060042 Bucharest, Romania; gabriel.dobri@upb.ro (G.D.); alexandrabanu14@yahoo.com (A.B.); 3 National Center for Micro and Nanomaterials, National University of Science and Technology POLITEHNICA Bucharest, 060042 Bucharest, Romania; truscaroxana@yahoo.com

**Keywords:** biomass ash, thermal power plant ash, supplementary cementitious materials, Portland cement, restoration mortars, built heritage, sustainable construction, circular economy

## Abstract

The construction sector is under increasing pressure to reduce natural-resource consumption and the environmental impact associated with Portland cement production. In this context, ashes generated by biomass combustion and thermal power plants may represent secondary resources for mineral binder-based construction materials, provided that their characteristics and performance are adequately established. This study comparatively characterized sunflower husk ash (SHA) and thermal power plant electrofilter ash (EFA) from Romanian sources. The experimental program comprised scanning electron microscopy, energy-dispersive X-ray spectroscopy, X-ray diffraction, pH and electrical-conductivity measurements, and preliminary sieve-based dimensional screening. The two ashes exhibited substantially different profiles. SHA was heterogeneous, strongly alkaline, highly conductive, and rich in K-, Ca-, Mg-, sulphate-, chloride-, and phosphate-bearing constituents, whereas EFA showed a predominantly silico-aluminous and iron-bearing profile, lower conductivity, and crystalline phases including quartz, hematite, and anhydrite. These results demonstrate that SHA and EFA cannot be treated as interchangeable ash-based materials and provide a basis for selecting subsequent investigations. They do not, however, establish pozzolanic activity, supplementary cementitious performance, or compatibility with specific construction applications. Validation in cementitious or lime-based systems must therefore include reactivity, workability, mechanical performance, dimensional stability, durability, and soluble-salt-related testing.

## 1. Introduction

The construction industry is one of the sectors with the highest consumption of natural resources, with Portland cement being the main binder used worldwide for the production of concrete, mortars, and numerous mineral-based composite materials. Although the technical performance of Portland cement has supported the development of modern infrastructure, its manufacturing process involves high energy consumption and significant carbon dioxide emissions, generated both by fuel combustion and by limestone decomposition during clinker production. The literature estimates that cement production contributes approximately 7–8% of global CO_2_ emissions, which highlights the need to reduce clinker content and identify alternative materials with lower environmental impact [1,2].

In this context, the use of supplementary cementitious materials represents one of the most important directions for innovation in sustainable construction. Ashes, slags, natural pozzolans, and certain mineral wastes can contribute to reducing cement consumption, valorizing residual material flows, and developing construction materials with properties adapted to different applications. However, the efficiency of these materials depends on their chemical composition, mineralogical phases, fineness, morphology, alkalinity, and behavior when incorporated into mineral binder-based systems. Therefore, the detailed characterization of such wastes is an essential step before proposing their use as partial cement replacements or functional additions.

Ash originating from thermal power plants, particularly fly ash collected by electrostatic precipitators, is one of the most widely known and studied by-products used in the field of construction materials. Due to its silico-aluminous composition and potential pozzolanic activity, it has frequently been investigated as a supplementary cementitious material in concrete and mortars, with effects on workability, mechanical strength development, and durability. However, in the context of European decarbonization policies and Romania’s commitments regarding the gradual phase-out of electricity production based on coal and lignite, the future availability of thermal power plant ash may become limited. This perspective requires both a better valorization of existing sources and stockpiles and the identification of alternative materials that could fulfill similar functions in cement-based composites [3,4,5].

In parallel, ashes resulting from the combustion of agricultural biomass represent a category of waste with technical and environmental potential, especially in countries or regions with significant agricultural activity. Sunflower husk ash is such a material, resulting from the energy recovery of a plant-based residue available in important agricultural areas. Unlike conventional fly ash from thermal power plants, biomass ashes generally show higher variability, determined by the type of biomass, combustion conditions, process temperature, collection method, and possible post-treatment procedures. Existing studies indicate that certain agricultural ashes, including those obtained from sunflower husks or other plant residues, may be considered as partial cement replacements; however, their use requires a careful evaluation of chemical composition, crystalline phases, and physical characteristics [6,7].

In Romania, the valorization of ashes obtained from local sources may have a dual importance. On the one hand, it can contribute to reducing the disposal of industrial and agricultural wastes, while on the other hand, it may support the development of construction materials adapted to regional resources. In the present study, the thermal power plant ash was collected from an electrostatic precipitation system located in the energy-producing area of Oltenia, Romania, while the sunflower husk ash originated from the Galați–Brăila region. The sunflower husk ash was analyzed in its raw form after drying at 105 °C. The selection of these materials allows a comparison between an industrial by-product with already recognized relevance in the field of cementitious materials and an agricultural biomass waste with potential for valorization within a circular economy framework.

Beyond conventional applications in concrete and mortars, these materials may warrant future investigation in repair, decorative, and conservation-related binder systems. However, compatibility with historic substrates cannot be inferred from ash characterization alone and requires dedicated testing of properties such as soluble salt release, porosity, permeability, mechanical compatibility, and interaction with lime-based binders.

Research on biomass ashes used in lime mortars suggests that such additions may have potential in the formulation of lower-impact materials; however, their applicability must be assessed according to the specific properties of each type of ash [8,9].

Therefore, this paper aims to comparatively characterize sunflower husk ash and thermal power plant ash collected from Romanian sources in order to assess their potential use as secondary materials in construction applications.

The experimental characterization includes morphological analysis by scanning electron microscopy, chemical composition assessment by energy-dispersive X-ray spectroscopy, crystalline phase identification by X-ray diffraction, as well as pH, electrical conductivity, and preliminary sieve-based dimensional screening determined by sieving.

The correlation of these results enables the identification of differences between the two ashes and provides a preliminary basis for selecting future experimental investigations in cementitious and lime-based systems. The present study does not aim to demonstrate supplementary cementitious performance or application-specific compatibility; these aspects require subsequent validation through binder-level testing.

## 2. Materials and Methods

The experimental program was designed to compare two ash-based residues of different origin: one agricultural biomass ash and one industrial ash collected from a thermal power plant. The approach was based on the characterization of the materials prior to their incorporation into cementitious or lime-based mixtures.

This stage is essential because the suitability of ash residues for construction applications depends on their intrinsic properties, including morphology, chemical and mineralogical composition, alkalinity, soluble ionic content, and granulometric distribution.

The methodology followed a comparative characterization strategy. The sunflower husk ash was selected as a representative agricultural biomass residue, while the thermal power plant ash was selected as an industrial by-product traditionally associated with supplementary cementitious applications.

Both materials were analyzed without chemical activation or blending with other binders, as presented in Table 1. The purpose was not to define an optimized mortar or concrete recipe at this stage, but to establish the material characteristics that may indicate possible valorization pathways in future binder hybrid systems.

### 2.1. Materials

The sunflower husk ash (SHA) was supplied by S.C. Prutul S.A., Galați, Romania, where sunflower husks are used as biomass fuel. According to information provided by the supplier, the biomass consisted exclusively of sunflower husks, which are processed into pellets prior to energetic valorization. Detailed operational parameters of the combustion process, including combustion temperature and furnace operating conditions, were not disclosed by the industrial supplier and were therefore unavailable for the present study. The investigated SHA originated from a single supplied batch.

Due to its agricultural origin and combustion-based formation, SHA may contain mineral phases and oxides derived from the inorganic fraction of the biomass, together with possible unburned residues depending on the combustion conditions [10,11,12]. Since the chemical and mineralogical composition of biomass ashes can vary significantly with the type of biomass, combustion temperature, residence time, and collection method, the material was considered a source-specific residue requiring direct experimental characterization.

The second investigated material was thermal power plant electrofilter ash, hereafter referred to as EFA (Electrofilter Fly Ash). The EFA originated from the Ișalnița Thermal Power Plant, Dolj County, Romania, where lignite is used as the principal solid fuel. According to the technical documentation of the Ișalnița generating units, the 510 t/h Benson boilers are equipped with fan mills in which lignite is ground and dried before the resulting pulverized lignite is conveyed to the boiler burners.

Flue-gas dedusting is subsequently performed by electrostatic precipitators, with two electrostatic precipitators installed for each boiler. Therefore, the investigated EFA is associated with pulverized-lignite combustion and electrostatic collection rather than with fluidized-bed combustion. The investigated material corresponds to the fine ash fraction collected by the electrostatic precipitation system and is distinct from the coarser ash stream separately discharged by the power plant [13].

However, their actual suitability as supplementary cementitious materials depends on their chemical composition, mineralogy, fineness, and possible content of crystalline or unburned phases [14,15].

The selection of SHA (Sunflower Husk Ash) and EFA (Electrofilter Fly Ash) allows a comparison between two different categories of ash residues. SHA represents an agricultural biomass-derived ash with potential relevance as an alternative local resource, while EFA represents an industrial ash historically linked with cement and concrete applications.

This comparative approach is also relevant in the context of the expected reduction in the availability of coal-combustion by-products, as the energy sector gradually transitions toward lower-carbon sources. Therefore, the study provides a preliminary basis for assessing whether agricultural biomass ashes may represent complementary or alternative resources for construction materials, particularly in regions where such residues are locally available.

A limitation of the present study is that each investigated ash originated from a single source and a single batch. Therefore, the results should be regarded as source- and batch-specific and should not be generalized to all sunflower husk ashes or coal-derived electrofilter ashes. Further investigations involving multiple production batches and different operating conditions are required to evaluate the variability and broader representativeness of these materials.

### 2.2. Sample Preparation

For the physical, chemical, and mineralogical analyses, SHA and EFA were stored separately under dry laboratory conditions at an approximately constant temperature of 20 °C in order to minimize exposure to moisture and contamination. The two materials were not mixed at any stage of the experimental program. Each material originated from a single batch. Prior to subsampling, each ash was manually homogenized separately to reduce segregation between coarse and fine particles. Representative subsamples were subsequently taken from the homogenized batches for SEM–EDS, XRD, pH, electrical conductivity, and particle-size analyses.

SHA was oven-dried at 105 °C for 24 h prior to characterization because the as-received material showed a tendency to absorb moisture and form agglomerates during storage. Drying was therefore applied to obtain a stable and reproducible powder condition prior to subsampling. In contrast, EFA remained dry, free-flowing, and finely divided under the laboratory storage conditions and was therefore characterized in its as-received state without additional oven drying. The moisture content of EFA was not independently determined.

The prepared subsamples were then used for preliminary sieve-based dimensional screening analysis by sieving, pH and electrical conductivity measurements, scanning electron microscopy, energy-dispersive X-ray spectroscopy, and X-ray diffraction analysis. The same sample codes, SHA (Sunflower Husk Ash) and EFA (Electrofilter Fly Ash), were maintained for all experimental procedures and result interpretation.

### 2.3. Characterization Methods

The characterization methodology was designed to obtain complementary information regarding the physical, chemical, mineralogical, and surface-related properties of the investigated ashes. Since ash-based residues may present significant variability depending on the raw material source, combustion conditions, collection system, and post-treatment procedures, a multi-technique approach is required before any construction-related application can be proposed [16,17].

Previous studies on biomass ashes and fly ash have shown that their potential use in cementitious or lime-based systems cannot be evaluated only through one parameter, but requires the correlation of morphology, elemental composition, crystalline phases, alkalinity, soluble ionic content, and preliminary sieve-based dimensional screening.

In the present study, scanning electron microscopy was used to observe the morphology and surface characteristics of the particles, while energy-dispersive X-ray spectroscopy was applied to determine the elemental composition of the investigated ashes. X-ray diffraction was used to identify the main crystalline phases and to provide information regarding the mineralogical nature of the materials.

In addition, pH and electrical conductivity measurements were performed in order to assess the alkalinity and soluble ionic content of the ashes, which are relevant parameters for their interaction with cementitious, lime-based, or hybrid binder systems. Preliminary sieve-based dimensional screening was performed by sieving, as the granulometric characteristics may influence water demand, packing behavior, filler effect, and the practical feasibility of using the ash in mortars, concrete, or decorative materials [16,17].

A similar integrated characterization strategy has been reported in studies dealing with agricultural biomass ashes. Quaranta et al. [18] characterized sunflower husk ash using microscopy, SEM–EDS, XRD, particle size analysis, pH, and electrical conductivity measurements, showing that these methods are useful for assessing the feasibility of incorporating such residues into ceramic or mineral-based products. Kalembkiewicz et al. [16] also used physicochemical and SEM–EDS analyses to evaluate biomass ash, reporting alkaline behavior, irregular particle morphology, and variable oxide composition, which supported the need for detailed characterization prior to proposing applications [16,18].

For coal fly ash and thermal power plant ash, characterization is also essential because standards and technical guidelines generally evaluate such materials through chemical and physical requirements, including oxide composition, fineness, moisture content, loss on ignition, and performance-related indicators. EN 450-1 specifies requirements for the chemical and physical properties of siliceous fly ash used as a type II addition in concrete, while ASTM C618-based discussions emphasize the importance of parameters such as the sum of SiO_2_, Al_2_O_3_, and Fe_2_O_3_, loss on ignition, fineness, and moisture content.

Therefore, although the present study does not aim to certify the investigated ashes according to these standards, the selected characterization methods provide a preliminary basis for assessing their compatibility with construction applications [15,19].

#### 2.3.1. Scanning Electron Microscopy SEM

Scanning electron microscopy was used to investigate the morphology and surface characteristics of the investigated ashes. SEM analysis is commonly applied in the characterization of biomass ashes and fly ashes because it allows the observation of particle shape, surface texture, porosity, agglomeration tendency and the presence of spherical, angular, porous or partially burned particles.

For SEM examination, a small amount of each ash was placed on double-sided conductive carbon adhesive tabs mounted on metallic specimen holders. The particles were gently distributed to obtain a sparse layer and were subsequently coated with a conductive metallic layer using a Quorum Technologies Q150R sputter coating system under vacuum conditions, with a chamber pressure of approximately 1 × 10^−3^ mbar. SEM observations were performed using a Quanta Inspect scanning electron microscope (FEI Company, Hillsboro, OR, USA), operated at an accelerating voltage of 30 kV and a spot size of 3.5, at magnifications ranging from 100× to approximately 50,000×.

A total of 34 micrographs was acquired for each ash. The images presented in the manuscript were selected because they contained dimensional measurements and illustrated the principal morphological features observed during the examination; they were not considered statistically representative of the entire ash batch.

These features are relevant for construction applications, as particle morphology may influence water demand, packing density, workability and the filler effect in cementitious or lime-based systems [16,18].

Previous studies have used SEM to characterize sunflower husk ash and other biomass ashes, reporting heterogeneous morphologies, irregular particles, agglomerates and variable particle sizes depending on the combustion process and ash source. SEM has also been widely used for thermal power plant ashes, where fly ash particles are often described as spherical, glassy, porous or irregular mineral fragments, depending on fuel type, combustion conditions and collection system [20].

#### 2.3.2. Energy-Dispersive X-Ray Spectroscopy EDS

Energy-dispersive X-ray spectroscopy was used together with SEM to determine the elemental composition of the investigated ashes and to support the interpretation of their morphological features.

EDS is commonly applied in the characterization of biomass ashes and fly ashes because it provides localized information regarding the chemical composition of selected particles or analyzed areas. Previous studies have used SEM–EDS to evaluate the heterogeneity of biomass ash particles and to identify major elements such as Si, Al, Ca, Mg, K, P, S and Fe, which are relevant for assessing their potential use in mineral binder-based systems [16,18,21].

For sunflower husk ash, Quaranta et al. used SEM–EDS, XRD, particle size analysis, pH and electrical conductivity measurements to characterize ash obtained from sunflower husk combustion, showing that this material requires source-specific evaluation before reuse in mineral-based products.

For coal fly ash, Kutchko and Kim used SEM–EDS to investigate the morphology and composition of Class F fly ashes from pulverized-coal power plants, reporting alumino-silicate and iron-rich particles with variable chemical characteristics. These studies highlight the relevance of EDS for correlating particle morphology with local chemical composition in ash-based materials [16,21].

EDS analyses were performed using area-based measurements rather than individual point analyses. For each investigated ash, five EDS analyses were performed on randomly selected analysis fields. The measurements were performed as area analyses rather than isolated single-point analyses. The reported elemental values represent the mean ± standard deviation of the five measurements.

The reported spectra were obtained at an accelerating voltage of 30 kV, with a specimen tilt of 0° and a take-off angle of 35.84°. A SUTW Sapphire detector was used, with a reported energy resolution of approximately 133.9 eV. Standardless EDAX ZAF quantification with elemental normalization was applied. The reported live-second (Lsec) values were 95 for SHA and 80 for EFA.

#### 2.3.3. X-Ray Diffraction XRD

X-ray diffraction was used to identify the main crystalline phases present in the investigated ashes. This method is relevant for ash characterization because the mineralogical composition strongly influences the possible behavior of ash residues in cementitious, lime-based or hybrid binder systems.

While EDS provides information on elemental composition, XRD allows the identification of crystalline compounds such as silicates, oxides, carbonates, phosphates, sulphates or iron-bearing phases.

Previous studies have shown that biomass ashes may contain complex mineral assemblages, depending on the type of biomass, combustion temperature and ash collection conditions. Vassilev et al. described biomass ashes as materials with variable phase-mineral composition, including associations related to silicates, carbonates, phosphates, sulphates, chlorides and glassy phases.

In the case of sunflower husk ash, Quaranta et al. used XRD together with SEM–EDS, pH, conductivity and particle size analysis, identifying phases such as MgCa(CO_3_)_2_, MgO, CaSiO_3_, Mg_2_P_2_O_7_ and K_2_Si_4_O_9_.

For fly ash from thermal power plants, XRD is commonly used to identify crystalline phases associated with the silico-aluminous fraction. Gomes et al. reported that silicoaluminous fly ashes typically contain crystalline phases such as α-quartz, mullite, hematite and magnetite within an aluminosilicate glass matrix.

Therefore, XRD analysis is essential for evaluating whether the investigated electrofilter ash presents mineralogical features comparable to conventional coal fly ash. The XRD results were further correlated with SEM morphology, EDS elemental composition, pH, electrical conductivity and preliminary sieve-based dimensional screening. X-ray diffraction (XRD) analysis was performed using a RIGAKU SmartLab 3 kW high-resolution X-ray diffractometer (Rigaku Corporation, Tokyo, Japan) for thin-film analysis.

#### 2.3.4. pH Measurement

For pH measurements, 10 g of each ash was dispersed in 100 mL of distilled water. The SHA mass corresponded to the material previously oven-dried at 105 °C for 24 h, whereas EFA was used in its as-received state. The suspensions were agitated for 30 min in order to promote contact between the solid material and the aqueous medium. Subsequently, the suspensions were filtered and decanted before measurement.

The pH was determined using a Hanna Instruments pH meter at a laboratory temperature of approximately 22 °C. Measurements were recorded at four contact times, designated T0, T1 (1 h), T2 (2 h), and T3 (3 h).

#### 2.3.5. Electrical Conductivity

Electrical conductivity was determined using aqueous suspensions prepared according to the same procedure described for the pH measurements. Thus, 10 g of each ash was dispersed in 100 mL of distilled water, agitated for 30 min, and subsequently filtered and decanted before measurement. Conductivity was recorded at T0, T1 (1 h), T2 (2 h), and T3 (3 h) at approximately 22 °C using a Hanna Instruments conductivity meter. The results were expressed of course in mS/cm.

Previous studies on sunflower husk ash and biomass ashes have included electrical conductivity as part of the physicochemical characterization of the material. Quaranta et al. reported high electrical conductivity for sunflower husk ash, together with alkaline pH, indicating the release of soluble alkaline species in distilled water.

Kalembkiewicz et al. also emphasized that biomass ash may contain alkaline compounds and soluble mineral constituents, which justify the use of aqueous-contact tests before proposing reuse applications. For fly ash, aqueous dispersion and leaching behavior are also relevant, as soluble ions and alkaline pH may influence the interaction of the ash with water and binder systems [16,18,22].

#### 2.3.6. Sieve-Based Dimensional Screening

A preliminary sieve-based dimensional screening was performed to obtain an indication of the coarse and fine fractions present in the investigated ashes. A 10 g sample of each ash was tested using a Fritsch Analysette 3 PRO vibratory sieve shaker (Fritsch GmbH, Idar-Oberstein, Germany) equipped with Prüfsieb DIN 4188 sieves with aperture sizes of 0.200 mm and 0.090 mm. The analysis was performed for 20 min at an amplitude of 1.5 mm. SHA was tested after oven drying at 105 °C for 24 h, whereas EFA was analyzed in its as-received state.

Because only two sieve sizes and a single 10 g test were used for each material, the procedure was considered a preliminary dimensional screening rather than a complete particle-size distribution determination. Accordingly, parameters such as d10, d50, d90 or d95 could not be determined from the present measurements.

Previous studies on sunflower husk ash have also included particle size analysis as part of the physicochemical characterization of the material, while studies on fly ash emphasize the importance of fineness for its behavior as a supplementary cementitious material [3,18].

## 3. Results and Discussion

### 3.1. Morphological Characteristics SEM

In the present study, the SHA and EFA samples were prepared by placing a small amount of ash on double-sided conductive carbon adhesive tabs mounted on metallic SEM specimen holders. The particles were gently distributed on the adhesive surface using a laboratory spatula in order to obtain a sparse particle layer and avoid excessive accumulation.

Prior to SEM observation, the specimens were coated with a thin conductive metallic layer using a Quorum Technologies Ltd. Q150R sputter coating system, under vacuum conditions, with the chamber pressure reaching approximately 1 × 10^−3^ mbar during the preparation cycle.

The SEM micrographs revealed clearly different morphological characteristics for SHA and EFA. The investigated areas were examined at magnifications ranging from 100× to ~50,000×. For each ash, 34 SEM micrographs were acquired at different magnifications to document the morphological variability of the samples.

The micrographs presented in Figure 1 and Figure 2 were selected from this larger dataset because they included particle-size measurements performed during the SEM examination and allowed both particle morphology and dimensional features to be illustrated. The selected images therefore represent documented measurement fields and were not intended to imply statistical representativeness of the entire ash batch. The surface characteristics and morphology are presented in Table 2.

The SEM micrographs of thermal power plant ash revealed a heterogeneous morphology, characterized by sub-rounded, ovoid, irregularly rounded and locally angular particles. At lower magnifications, the material appeared as a mixture of individual coarse particles and finer fractions distributed between larger grains. Compared with sunflower husk ash, EFA showed a more rounded particle morphology; however, the particles were not predominantly smooth or glassy in the selected micrographs.

At medium and high magnifications, the surface of the particles appeared rough, granular and locally porous. Several particles exhibited visible cavities, microvoids and vesicular-looking areas, indicating a complex surface structure.

Fine particles were also observed attached to larger grains or distributed within agglomerated zones. These features suggest that the investigated electrofilter ash is not morphologically uniform and that its behavior in binder systems may depend on both its coarse and fine fractions.

In the literature, coal fly ash collected from thermal power plants is often described as containing spherical or glassy particles, including microspheres, plerospheres, porous microspheres and irregular mineral fragments. However, in the present EFA sample, the selected SEM images did not indicate a clear predominance of smooth glassy microspheres. Instead, the observed morphology was mainly represented by rough, porous, sub-rounded and agglomerated particles.

This difference highlights the importance of source-specific characterization, since the morphology of ash residues may vary depending on combustion conditions, collection system, fuel type and post-combustion handling. From a construction-material perspective, the rounded tendency of some EFA particles may contribute positively to packing and flow behavior. Nevertheless, the rough surface texture and visible porosity may increase water demand if the ash is incorporated directly into cementitious or lime-based mixtures.

Therefore, the morphological observations should be interpreted together with the EDS, XRD, pH, electrical conductivity and granulometric results before any conclusion regarding its suitability as a supplementary cementitious material is formulated. Although the predominance of rough, porous and irregular particles may resemble morphologies reported for some fluidized-bed combustion ashes, particle morphology alone was not used to infer the combustion technology of the investigated EFA. The documented origin of the sample indicates pulverized-lignite combustion followed by electrostatic collection. Therefore, the observed morphology is interpreted as a source-specific feature of the investigated lignite-derived electrofilter ash.

### 3.2. Elemental Composition EDS

In the present study, the elemental composition of the two samples was estimated using energy-dispersive X-ray spectroscopy (EDS). A minimum of three EDS measurements were performed for each ash on different selected surface areas in order to account, within the limitations of the method, for local compositional heterogeneity.

The spectra presented in Figure 3 and Figure 4 correspond to representative analyzed areas selected from these measurements. EDS was therefore used as a localized, semi-quantitative technique and not as a determination of bulk chemical composition. Carbon was excluded from the reported elemental quantification because the powders were mounted on carbon adhesive tape. The chemical composition was determined in the tables included in Table of Figure 3.

In the representative EDS areas analyzed for the present SHA sample, K and Ca were predominant, whereas Si and Al were not detected in the spectrum reported in Figure 3. Because EDS provides localized and semi-quantitative elemental information, this observation should not be interpreted as evidence for the complete absence of Si and Al from the bulk SHA material. Instead, the result indicates that the investigated regions were predominantly enriched in K, Ca, and Mg-bearing constituents rather than exhibiting the silico-aluminous composition typically associated with conventional coal fly ash.

For each investigated ash, five EDS analyses were performed on randomly selected analysis fields within the available SEM examination area. In contrast, EFA exhibited a predominantly silico-aluminous elemental profile. The reported mean values were 28.67 ± 2.69 wt.% Si and 16.49 ± 3.61 wt.% Al, together with 5.17 ± 1.03 wt.% Fe and 3.29 ± 1.35 wt.% Ca. Lower contents of Mg, K, Ti and S were also detected. These results refer to the five analyzed SEM–EDS areas and should not be interpreted as bulk chemical composition.

This composition is consistent with literature reports showing that biomass ashes, including sunflower-derived ashes, often contain significant amounts of alkaline and alkaline-earth elements such as K, Ca and Mg. Kalembkiewicz et al. [16] also noted that biomass ashes may contain high amounts of alkaline compounds, particularly calcium, magnesium, sodium and potassium oxides. Compared with previous work on sunflower husk ash, the present SHA sample shows a similar tendency toward a composition rich in alkaline and alkaline-earth elements. In the analyzed EDS field, Si and Al were below the detection capability of the reported measurement. This observation refers exclusively to the investigated EDS area and should not be interpreted as evidence of the general absence of Si and Al from the SHA batch.

Kutchko and Kim [21] reported that Class F fly ash samples were mainly composed of amorphous alumino-silicate spheres and smaller amounts of iron-rich particles, while other studies describe fly ash as a source of silica, alumina, iron and calcium compounds. Overall, in the reported EDS analysis field, K and Ca were the dominant detected metallic elements, while Si and Al were not detected. However, because EDS provides localized and semi-quantitative elemental information, this observation should not be interpreted as evidence of the general absence of Si and Al from the SHA batch. Consequently, the bulk silico-aluminous character of SHA cannot be established or excluded on the basis of the present EDS measurements alone. Its potential use in construction materials should therefore be evaluated carefully, especially in relation to its alkalinity, soluble salts, mineral phases and granulometric characteristics. Furthermore, the present SEM and EDS characterization concerns the ash particles prior to their incorporation into a binder. The microstructural behavior of the hardened binder cannot be inferred directly from these observations.

Recent investigations of hardened cement paste have demonstrated that microscale heterogeneity, including the spatial distribution of phases and porosity, can substantially influence the resulting mechanical response. Therefore, future work should extend the present characterization to cementitious and/or lime-based pastes containing SHA and EFA in order to investigate particle–matrix interactions, reaction products, pore structure, and microstructural heterogeneity after hardening [23].

### 3.3. Mineralogical Composition XRD

In the present study, XRD analysis was performed on the SHA and EFA samples in order to determine their crystalline phase composition and to support the interpretation of the EDS results. The obtained diffractograms were used to identify the main mineralogical phases and to compare the biomass ash with the electrofilter ash from the thermal power plant, as presented in Figure 5 and detailed in Table 3.

In addition to these phases, several candidate phases may also be present, including CaO (lime), calcium phosphates and calcium silicates, iron oxides, and phosphorus- or sulfur-containing oxides. However, these phases are likely present in low concentrations and, under the current experimental conditions, cannot be identified with certainty. For the EFA sample, the XRD pattern, together with the positions of the reference diffraction peaks, is shown in the Figure 6 and detailed in Table 4.

For this ash sample, the phases listed in Table 4 were identified with confidence. These phases represent the major crystalline constituents of the sample.

The XRD analysis was qualitative and was used for crystalline-phase identification only. No quantitative phase analysis or amorphous-fraction determination was performed. Consequently, the relative abundance of the identified phases and the reactive amorphous fraction cannot be established from the present XRD data, and the mineralogical results should not be interpreted as direct evidence of pozzolanic activity.

### 3.4. Water Behavior pH and Electrical Conductivity

For coal fly ash and thermal power plant ash, pH is also an important characterization parameter because it may vary depending on the chemical type of ash, calcium content, soluble salts and combustion conditions. Fly ash properties are known to differ according to origin and composition, which affects their possible reuse in cementitious and geotechnical applications. Therefore, pH measurements were included in the present study to compare the alkaline behavior of SHA and EFA and to support the interpretation of the EDS and XRD results [16,18]. In this case, pH evolution of the investigated ashes in distilled water is presented in Table 5 and in Figure 7 as well.

The pH results indicate that both investigated ashes exhibited alkaline behavior throughout the monitored contact period, although their evolution in time was different. For SHA, the pH increased from 13.33 ± 0.006 at T0 to 13.64 ± 0.025 after 1 h, followed by a slight decrease to 13.52 ± 0.036 after 2 h and a subsequent increase to 13.86 ± 0.006 after 3 h.

Despite the intermediate fluctuation, the results indicate an overall slight increasing tendency of pH during the investigated period, while maintaining a strongly alkaline character.

In contrast, EFA showed a decreasing pH tendency. The initial value of 11.71 ± 0.30 decreased to 10.85 ± 0.049 after 1 h and remained nearly stable at 10.84 ± 0.020 after 2 h, followed by a slight increase to 10.90 ± 0.012 after 3 h.

Thus, the main pH decrease occurred during the first hour of contact with water, after which only limited variations were observed. EFA nevertheless remained alkaline throughout the investigated period.

The different pH evolution of the two ashes further confirms their distinct aqueous behavior. The persistently high pH values recorded for SHA are consistent with the presence of K-, Ca-, and Mg-bearing constituents identified by EDS and with the alkaline mineral phases observed by XRD, whereas the lower and decreasing pH values of EFA indicate a comparatively less alkaline response under the investigated conditions.

Given that the observed differences are relatively small, a statistical analysis was required to determine whether they were significant. Individual *t*-tests confirmed that the differences between the groups were statistically significant. In addition, a one-way analysis of variance (ANOVA) was performed at a significance level of α = 0.05, and the results are presented in Table 6.

The ANOVA results indicate statistically significant differences among the measured values, demonstrating that the pH varies over time.

The post hoc Tukey test further revealed statistically significant differences between individual measurements, confirming that the observed phenomenon evolves over time, presented in Table 7.

For the EFA sample, the ANOVA results are presented in Table 8.

The ANOVA results revealed statistically significant differences, indicating that the pH changes over time. However, the Tukey post hoc test showed no statistically significant differences among the mean pH values measured at 1 h, 2 h, and 3 h, as presented in Table 9. The results were used to compare This finding suggests that the process is essentially completed within approximately 1 h, after which no significant changes are observed.

In the present study, electrical conductivity was determined using the aqueous preparations described in Section 2.3.5 (10 g of ash dispersed in 100 mL of distilled water). The suspensions were filtered and decanted before measurement, and conductivity values were recorded for both SHA and EFA at different contact times, and the results are presented in Table 10.

Therefore, the SHA mass corresponded to the oven-dried material, whereas the EFA mass corresponded to the as-received material stored under dry laboratory conditions. Since the moisture content of EFA was not independently determined, its mass cannot strictly be considered an oven-dry mass.

The results were used to compare the soluble ionic character of the two ashes and to support the interpretation of the pH, EDS and XRD data. The Tukey post hoc test identified two distinct groups.

The electrical conductivity results indicate markedly different aqueous behaviors for the two investigated ashes. SHA exhibited very high conductivity throughout the entire monitoring period. The conductivity increased slightly from 20.12 ± 0.332 mS/cm at T0 to 20.34 ± 0.226 mS/cm after 1 h, followed by a more pronounced increase to 21.11 ± 0.091 mS/cm after 2 h. At 3 h, the value remained nearly unchanged at 21.07 ± 0.173 mS/cm. Overall, SHA therefore showed a slight increasing tendency in electrical conductivity during the first 2 h, followed by stabilization.

EFA exhibited substantially lower conductivity values than SHA. The initial conductivity was 0.064 ± 0.002 mS/cm, increasing markedly to 0.77 ± 0.006 mS/cm after 1 h and only slightly to 0.79 ± 0.004 mS/cm after 2 h. At 3 h, a minor decrease to 0.78 ± 0.001 mS/cm was observed. These results indicate that the main increase in ionic release from EFA occurred during the first hour of contact with water, after which the conductivity remained practically stable.

The contrasting conductivity evolution confirms the different soluble ionic behavior of the two ashes. SHA maintained a very high ionic release throughout the investigated period, whereas EFA showed a much lower conductivity level and reached an approximately stable condition after the initial increase, as presented in Figure 8.

For the SHA sample, the ANOVA results are present in Table 11.

The ANOVA results revealed statistically significant differences among the group means, indicating that exposure time has a significant effect on electrical conductivity.

The Tukey post hoc test identified two distinct groups: the mean electrical conductivity values at 0 h and 1 h were not statistically different, and similarly, no statistically significant differences were observed between the mean values at 2 h and 3 h, as presented in Table 12.

These findings suggest that the process is initiated after approximately 2 h and progresses relatively slowly, as no significant changes in electrical conductivity occur within a one-hour interval.

For the EFA sample, the ANOVA results are presented in Table 13.

The ANOVA results revealed a statistically significant effect of contact time on the electrical conductivity of EFA (F(3,8) = 26,995.93, *p* < 0.001). The pronounced statistical effect was primarily associated with the marked increase from the initial measurement to the subsequent contact times. Although the conductivity values measured after 1 h varied only slightly, from 0.77 to 0.79 mS/cm, the low within-group variability resulted in statistically detectable differences among these time points. From a practical perspective, however, the major change occurred during the first hour, after which the conductivity remained within a narrow range of 0.77–0.79 mS/cm.

The Tukey post hoc test confirmed that the initial conductivity value differed significantly from all subsequent measurements, as presented above in Table 14. Statistically significant differences were also detected among the 1 h, 2 h and 3 h measurements; however, these differences corresponded to absolute changes of only 0.01–0.02 mS/cm. Therefore, while contact time had a statistically significant effect, the EFA conductivity showed only limited practical variation after the pronounced increase observed during the first hour.

### 3.5. Sieve-Based Dimensional Assessment

The preliminary sieve screening showed that 58.58% of the SHA sample and 51.33% of the EFA sample passed the 0.090 mm sieve. These results indicate that a substantial fraction of both investigated ashes consists of particles smaller than 90 µm.

This observation should be interpreted only as an indication of dimensional compatibility with the broader size domain of cementitious powders and not as evidence of an equivalent particle-size distribution. Ordinary Portland cement particles have been reported to extend over a broad particle-size range, approximately 1–100 µm, depending on grinding conditions and cement type [24,25]. Therefore, the fraction of the investigated ashes passing the 90 µm sieve presents a dimensional overlap with the upper part of the particle-size range encountered in conventional cement. However, the present sieve screening does not provide sufficient information to establish similarity with the complete cement particle-size distribution.

Furthermore, no conclusion regarding injectability can be derived from the present screening alone. The penetrability of particulate cementitious grouts depends not only on an upper particle-size threshold but also on the complete particle-size distribution, characteristic particle diameters such as d95, agglomeration, rheology and the relationship between particle size and crack or pore aperture [24,25,26]. Consequently, the present results are considered only a preliminary dimensional criterion, while a more resolved particle-size analysis and direct groutability testing are required before injectable-mortar performance can be assessed.

### 3.6. Integrated Assessment for Construction Applications

The comparative characterization demonstrates that SHA and EFA are distinct source-specific materials and cannot be considered equivalent. The observations reported here identify characteristics that must guide subsequent testing; they do not establish suitability for a construction application.

For SHA, the high alkalinity, very high electrical conductivity, and K-, sulphate-, and chloride-bearing constituents make soluble-ion release, leaching, efflorescence, and binder compatibility priority subjects for further study. Possible processing steps such as grinding, controlled sieving, or washing should be evaluated experimentally and cannot be recommended on the basis of the present data alone.

For EFA, the silico-aluminous and iron-bearing profile provides a rationale for testing it as a mineral addition in cementitious systems. Nevertheless, its amorphous content, pozzolanic reactivity, loss on ignition, water demand, and effects on fresh and hardened properties were not determined; therefore, neither supplementary cementitious performance nor an appropriate replacement level can be inferred.

Any investigation of either ash in repair, decorative, lime-based, or conservation-related materials must include application-specific performance and compatibility tests. In particular, compatibility with historic substrates cannot be inferred from the present ash characterization. In this case evidence-based assessment and priorities for subsequent testing are presented in Table 15.

## 4. Conclusions

This study demonstrated that sunflower husk ash (SHA) and thermal power plant electrofilter ash (EFA) from the investigated Romanian sources have substantially different physicochemical and mineralogical profiles and should not be treated as interchangeable ash-based materials. SHA exhibited heterogeneous morphology, strong alkalinity, very high electrical conductivity, and K-, Ca-, Mg-, sulphate-, chloride-, and phosphate-bearing constituents. By comparison, EFA presented a more pronounced silico-aluminous and iron-bearing profile, substantially lower conductivity, and crystalline phases including quartz, hematite, and anhydrite.

The reported characterization establishes the intrinsic differences between the two ashes but does not demonstrate their performance in a binder. In particular, the results do not establish pozzolanic activity, suitability as a supplementary cementitious material, injectability, durability, or compatibility with lime-based binders and historic substrates. The high alkalinity and ionic release observed for SHA identify soluble salts and related compatibility effects as matters requiring specific assessment rather than as confirmed application-level outcomes. The profile identified for EFA warrants further investigation of its behavior as a thermal-power-plant-derived mineral addition in cementitious systems.

The scope of the findings is limited by the use of one source and one batch for each ash, the semi-quantitative and localized nature of EDS analysis, and the preliminary particle-size assessment based on two sieve thresholds without a resolved particle-size distribution or repeated measurements. Consequently, the results should be interpreted as source-specific screening data and should not be generalized to all sunflower husk ashes or thermal power plant electrofilter ashes.

Future work should evaluate both materials in paste and mortar formulations through direct pozzolanic-reactivity testing and measurements of workability, setting behavior, mechanical strength, dimensional stability, porosity, permeability, and durability. For SHA, soluble-salt release, leaching, and efflorescence risk require particular attention. For conservation-related applications, compatibility with lime-based binders and representative historic substrates must be established separately before any practical use can be proposed.

The authors are grateful to the Romanian Government for providing access to the research infrastructure of the National Center for Micro and Nanomaterials through the National Program titled ”Installations and strategic Objectives of National Interest”.

## Figures and Tables

**Figure 1:**
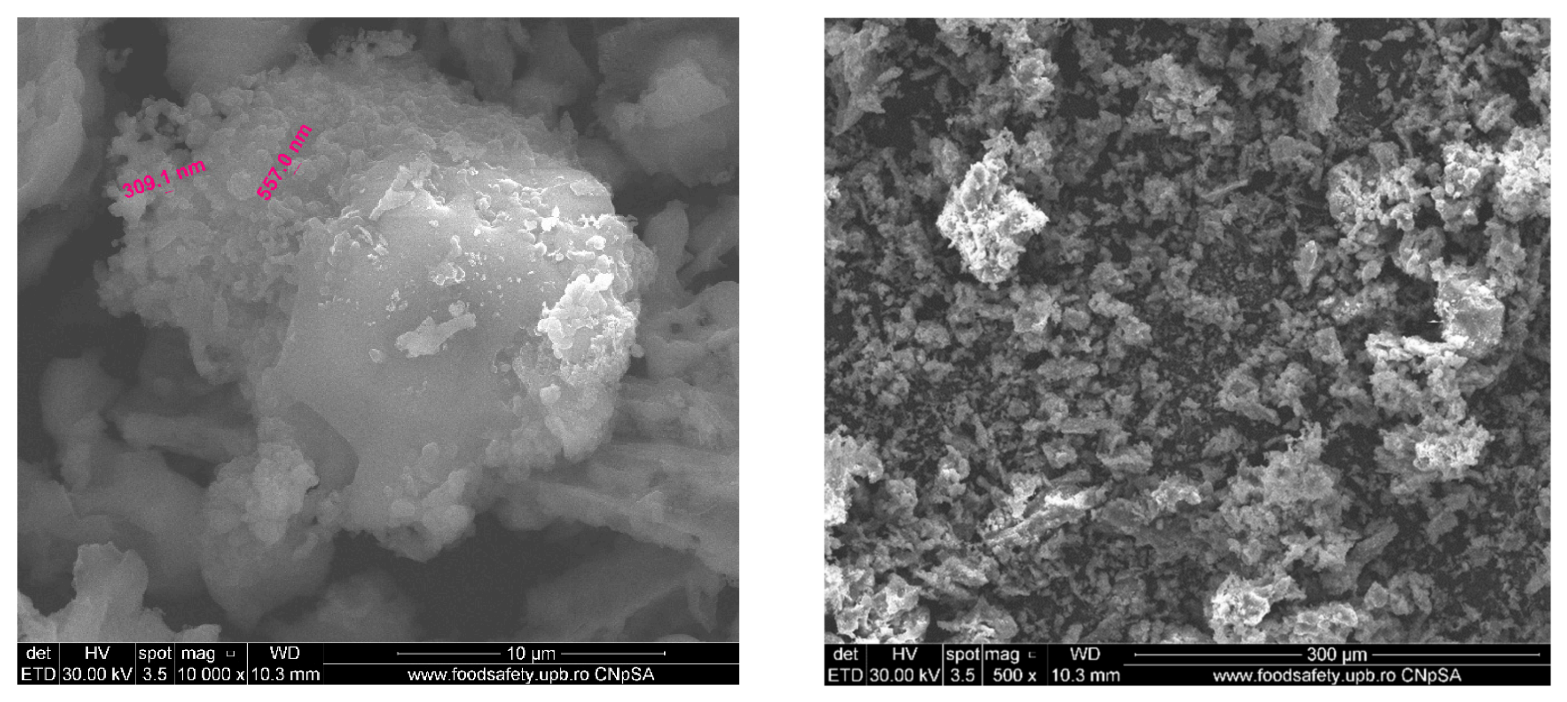
SEM morphology of sunflower husk ash.

**Figure 2:**
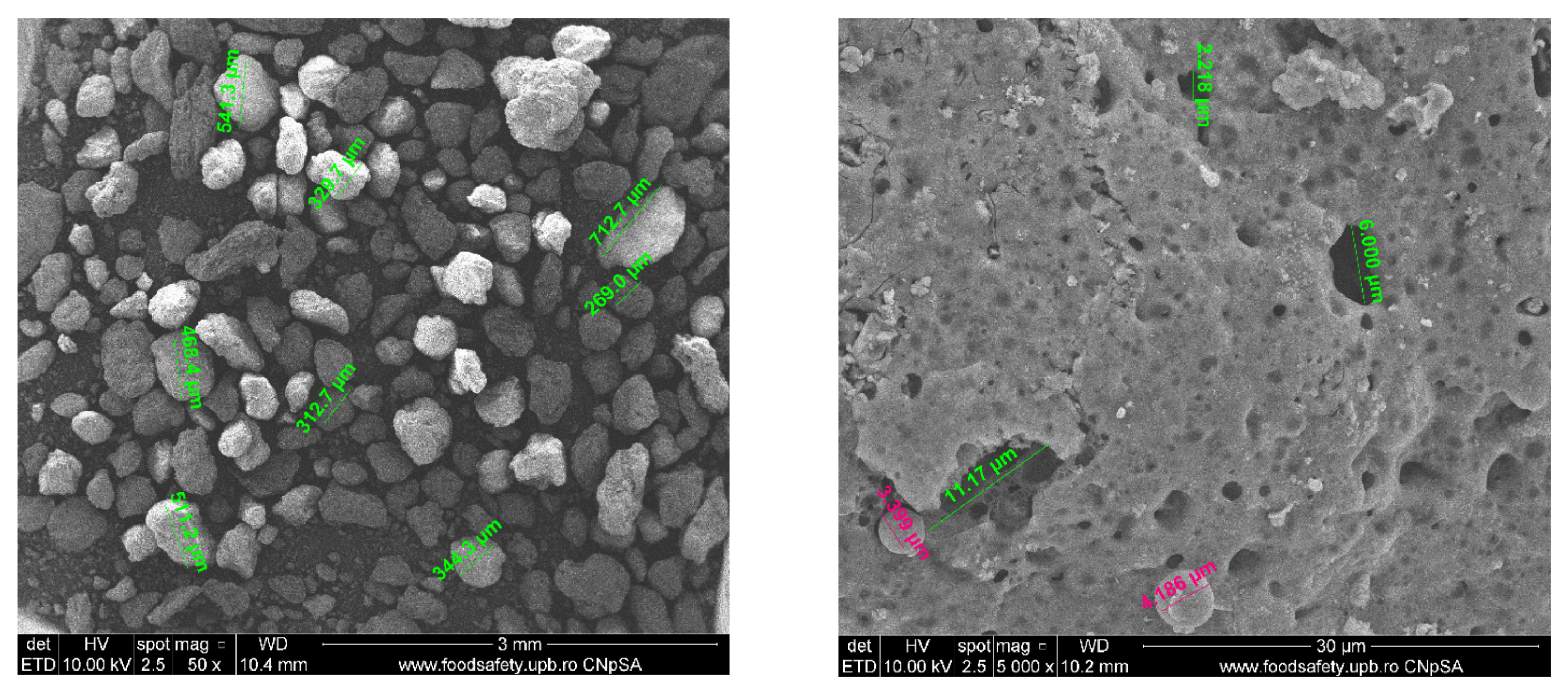
SEM morphology of thermal power plant ash.

**Figure 3:**
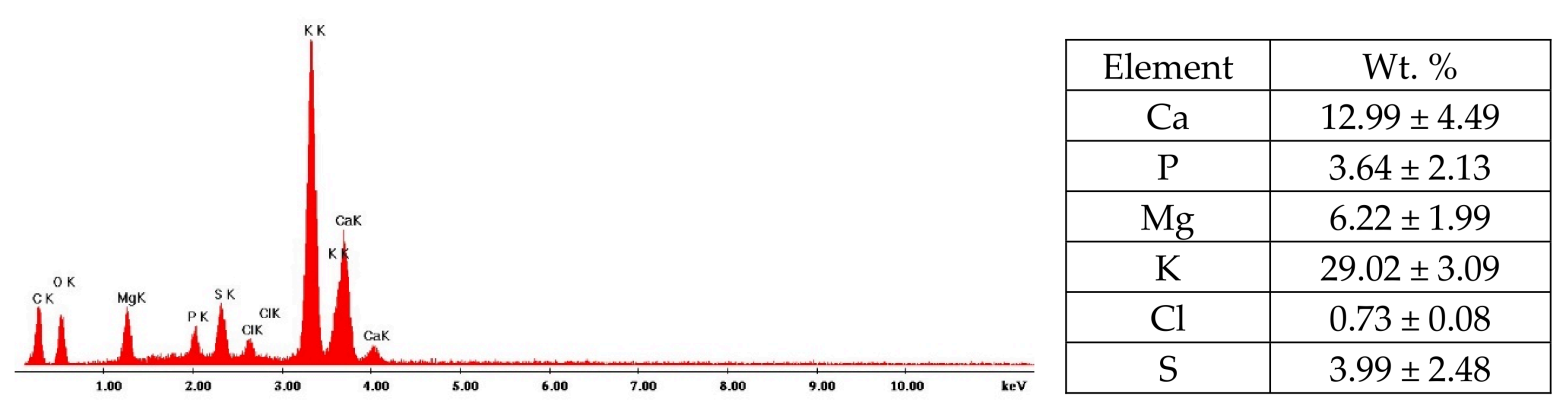
EDS analysis for SHA (Sunflower Husk Ash).

**Figure 4:**
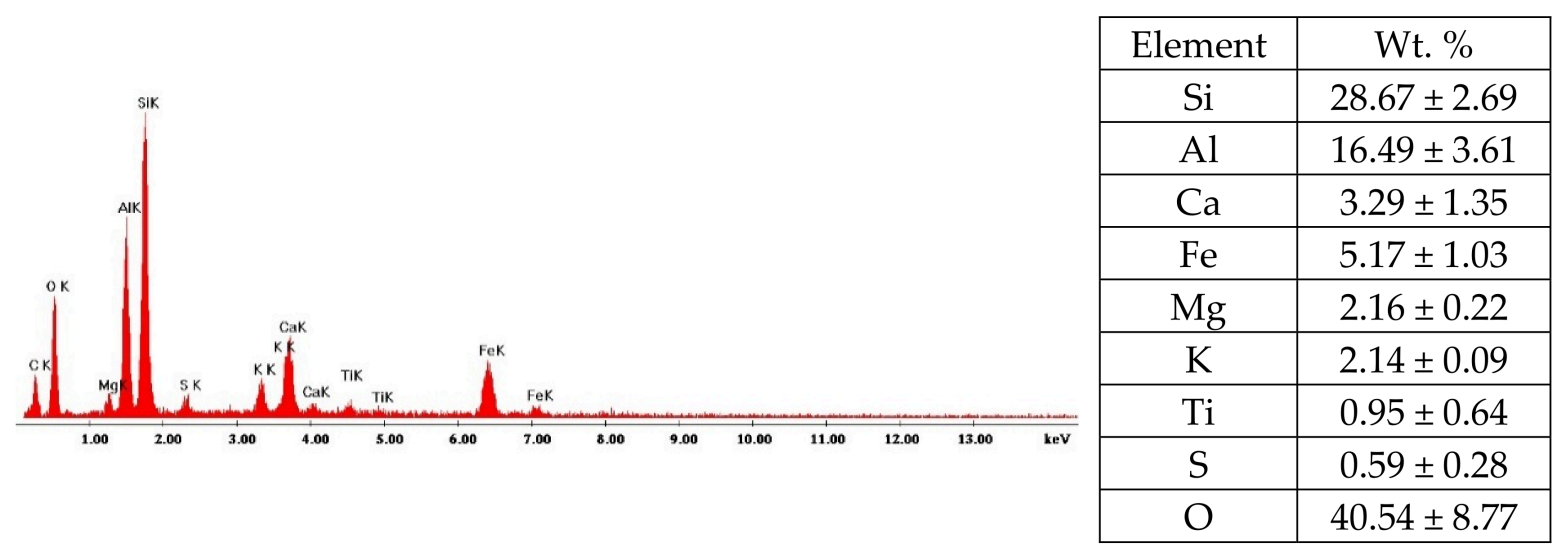
EDS analysis for EFA (Electrofilter Fly Ash).

**Figure 5:**
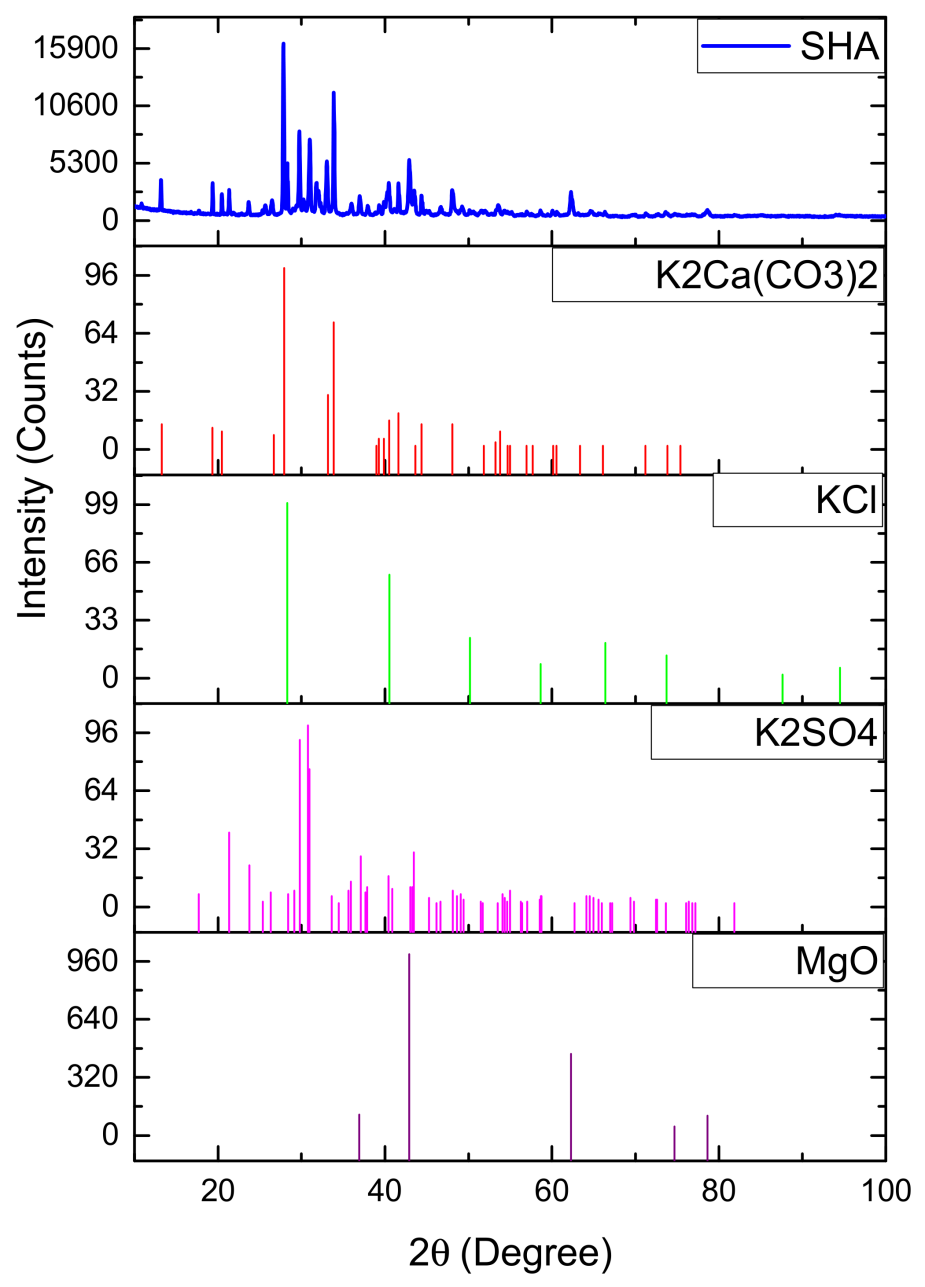
XRD pattern of SHA.

**Figure 6:**
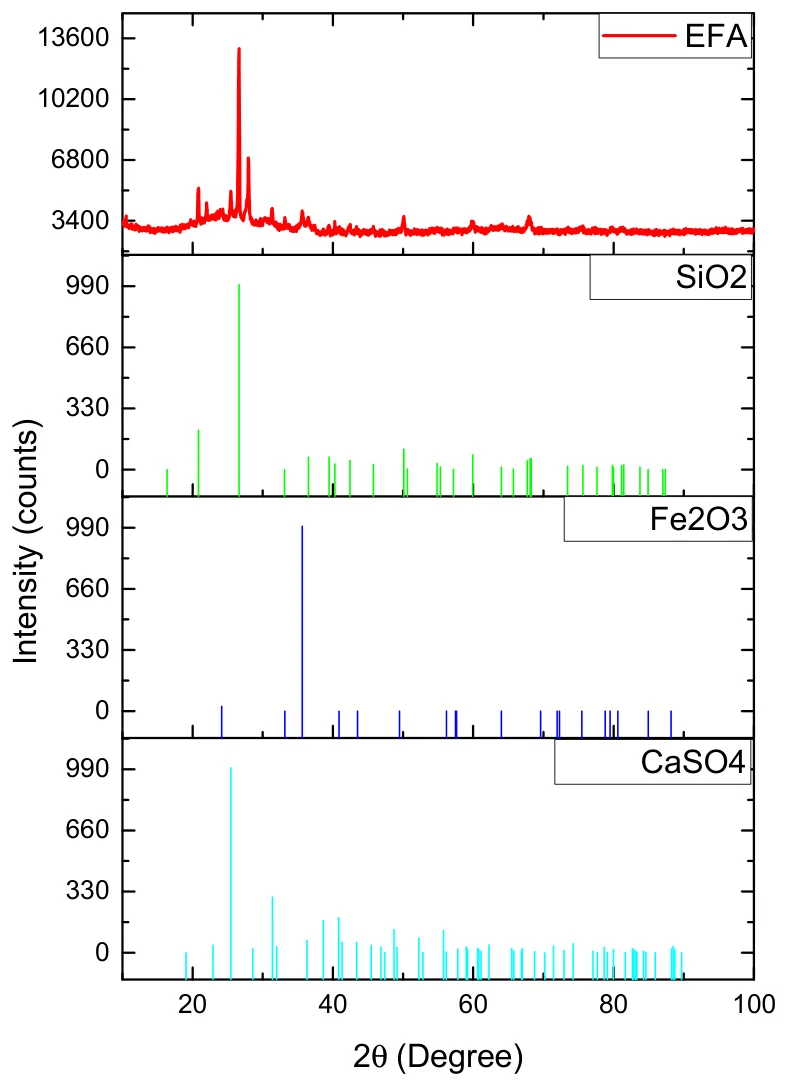
XRD pattern of EFA.

**Figure 7:**
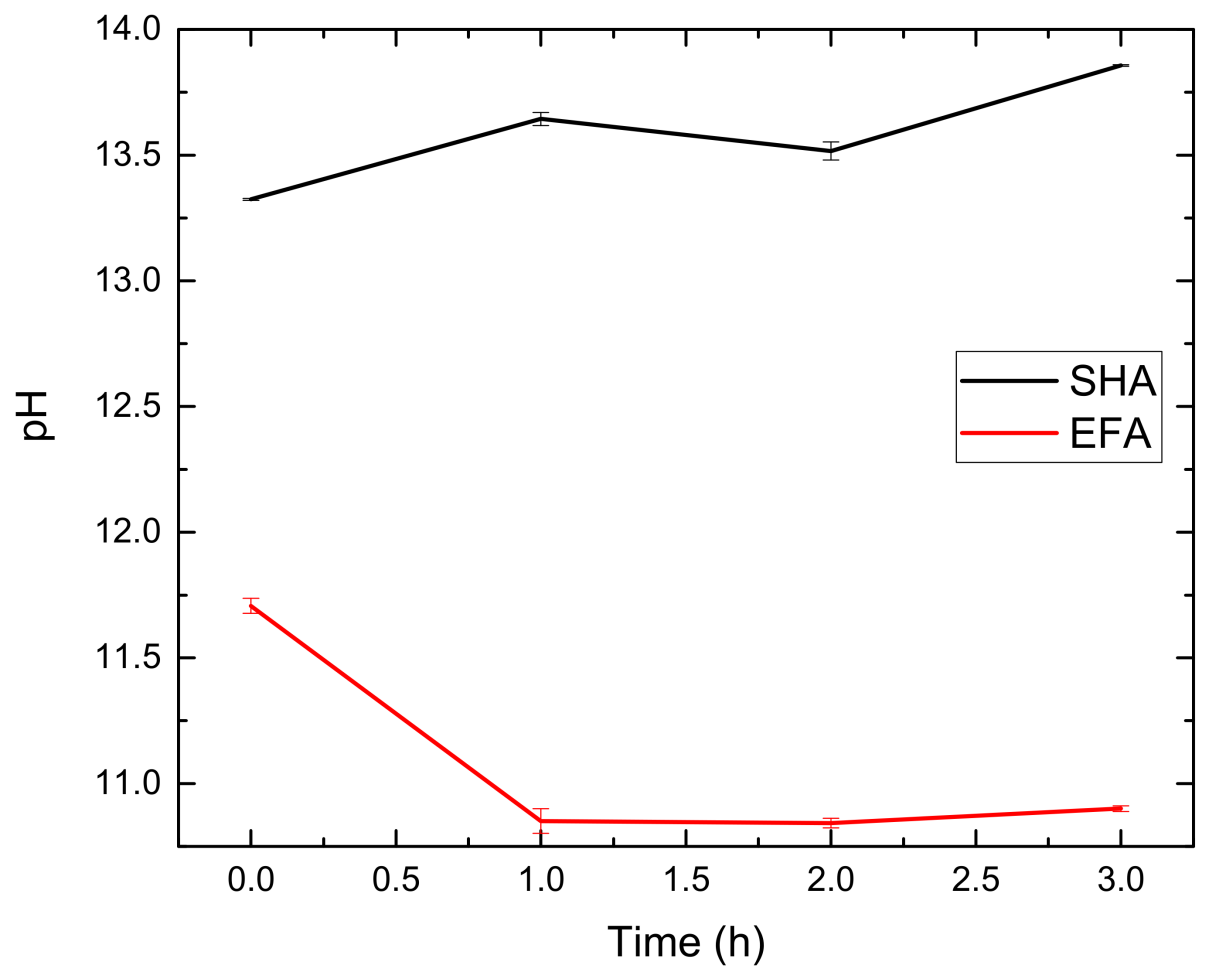
SHA and EFA in pH evolution.

**Figure 8:**
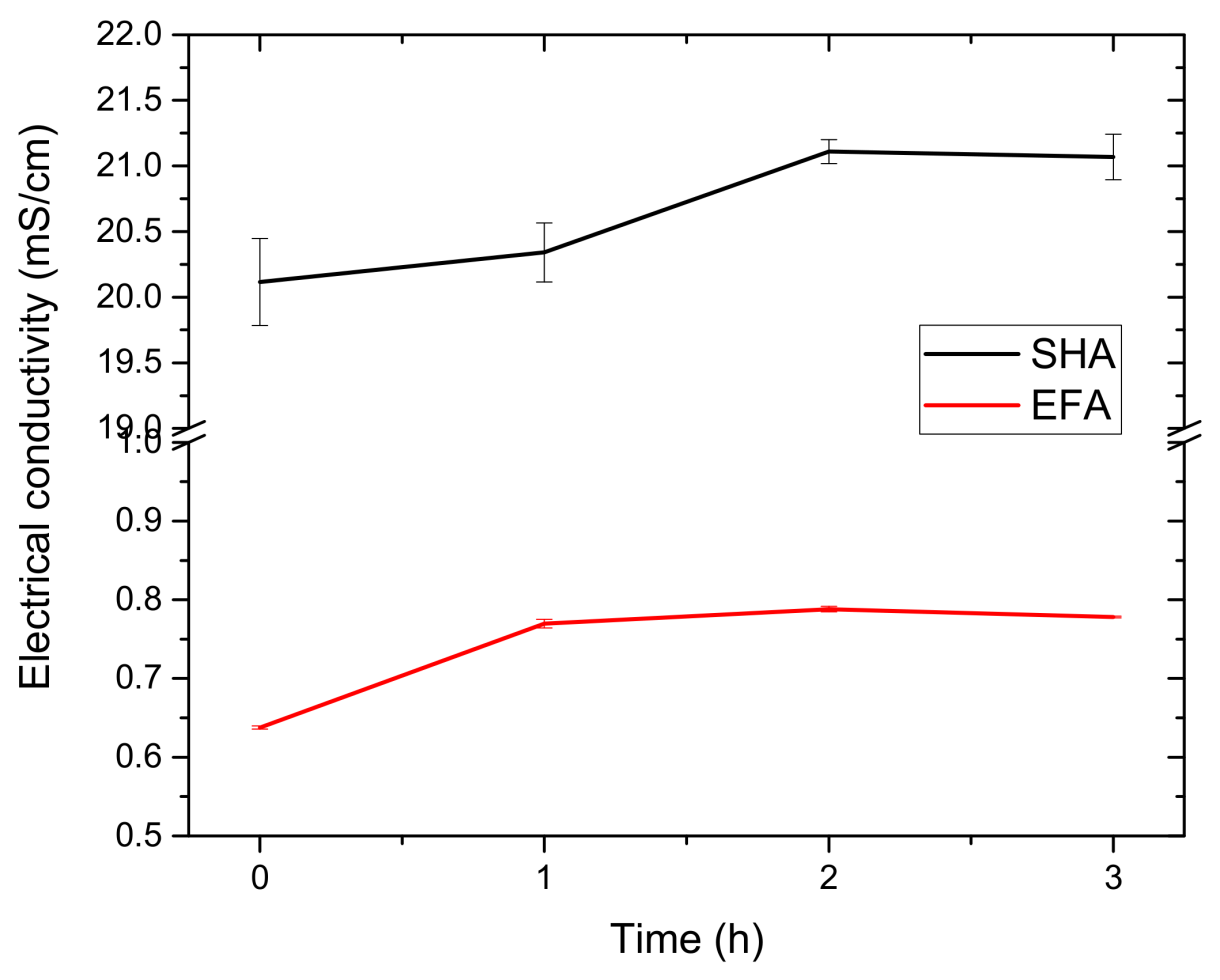
SHA and EFA in Electrical Conductivity evolution.

**Table 1:** The investigated materials.

Sample Code	Material	Origin	Collection Source	Preparation State
SHA (sunflower husk ash)	Sunflower husk ash	Galați–Brăila region, Romania	Collected from combustion furnaces	Dried at 105 °C
EFA (electrofilter ash collected from a thermal power plant)	Electrofilter ash from thermal power plant	Oltenia energy area, Romania	Collected from electrostatic precipitator	Analyzed in raw form

**Table 2:** Comparative SEM morphology features of the investigated ashes EFA.

Sample	Morphology	Surface Characteristics	Interpretation
SHA	Irregular, fragmented and locally rounded particles	Rough, heterogeneous surfaces with local porosity and fine adhered particles	May require granulometric optimization before use in binder systems
EFA	Sub-rounded, ovoid and locally angular particles	Rough, granular and porous surfaces with moderate agglomeration	Morphology suggests potential filler contribution, but suitability depends on chemical and mineralogical properties

**Table 3:** SHA sample presenting the crystalline phases unequivocally identified by X-ray diffraction (XRD).

Chemical Formula	Name	PDF No.	System	Space Group
K_2_Ca(CO_3_)_2_	Fairchildite	21-1287	hexagonal	P63/mmc
KCl	Sylvite	4-587	cubic	Fm-3m
K_2_SO_4_	Arcanite	24-703	orthorhombic	Pnma
MgO	Periclase	78-430	cubic	Fm-3m

**Table 4:** EFA sample presenting the crystalline phases assigned on the recorded XRD pattern and reference-pattern matching.

Chemical Formula	Name	PDF No.	System	Space Group
SiO_2_	Quartz	77-1060	triclinic	P1
Fe_2_O_3_	Hematite	73-603	rhombohedral	R-3c
Ca(SO_4_)	Anhydrite	72-503	orthorhombic	Amma

**Table 5:** pH evolution of the investigated ashes in distilled water.

Sample	T_0_	T_1_ (1 h)	T_2_ (2 h)	T_3_ (3 h)	Interpretation
SHA	13.33 ± 0.006	13.64 ± 0.025	13.52 ± 0.036	13.86 ± 0.006	Strongly alkaline
EFA	11.71 ± 0.30	10.85 ± 0.049	10.84 ± 0.020	10.90 ± 0.012	Alkaline

**Table 6:** ANOVA-pH-SHA.

Cases	Sum of Squares	df	Mean Square	F	*p*	η^2^	ω^2^
Time	0.444	3	0.148	296.3	<0.001	0.991	0.987
Residuals	0.004	8	5.000 × 10^−4^				

Note. Type III Sum of Squares.

**Table 7:** Post Hoc Comparisons–Time-SHA.

		Mean Difference	SE	df	t	*p*_Tukey_
0H	1H	−0.317	0.018	8	−17.34	<0.001
	2H	−0.193	0.018	8	−10.59	<0.001
	3H	−0.530	0.018	8	−29.03	<0.001
1H	2H	0.123	0.018	8	6.755	<0.001
	3H	−0.213	0.018	8	−11.68	<0.001
2H	3H	−0.337	0.018	8	−18.44	<0.001

Note. *p*-value adjusted for comparing a family of 4 estimates.

**Table 8:** ANOVA-pH-EFA.

Cases	Sum of Squares	df	Mean Square	F	*p*	η^2^	ω^2^
Time	1.600	3	0.533	567.6	<0.001	0.995	0.993
Residuals	0.008	8	9.395 × 10^−4^				

Note. Type III Sum of Squares.

**Table 9:** Tukey Post Hoc Comparisons of EFA pH between contact times.

		Mean Difference	SE	df	t	*p*_Tukey_
0H	1H	0.855	0.025	8	34.18	<0.001
	2H	0.864	0.025	8	34.51	<0.001
	3H	0.806	0.025	8	32.21	<0.001
1H	2H	0.008	0.025	8	0.333	0.986
	3H	−0.049	0.025	8	−1.971	0.274
2H	3H	−0.058	0.025	8	−2.304	0.176

Note. *p*-value adjusted for comparing a family of 4 estimates.

**Table 10:** Electrical conductivity evolution of the investigated ashes in distilled water.

Sample	T_0_	T_1_ (1 h)	T_2_ (2 h)	T_3_ (3 h)	Interpretation
SHA	20.12 ± 0.332	20.34 ± 0.226	21.11 ± 0.091	21.07 ± 0.173	Very high ionic release
EFA	0.064 ± 0.002	0.77 ± 0.006	0.79 ± 0.004	0.78 ± 0.001	Low to moderate ionic release

**Table 11:** ANOVA-C(mS/cm)-SHA.

Cases	Sum of Squares	df	Mean Square	F	*p*	η^2^	ω^2^
Time	2.301	3	0.767	15.41	0.001	0.853	0.783
Residuals	0.398	8	0.050				

Note. Type III Sum of Squares.

**Table 12:** Tukey Post Hoc Comparisons of SHA electrical conductivity between contact times.

		Mean Difference	SE	df	t	*p*_Tukey_
0H	1H	−0.225	0.182	8	−1.233	0.625
	2H	−0.994	0.182	8	−5.455	0.003
	3H	−0.953	0.182	8	−5.230	0.004
1H	2H	−0.769	0.182	8	−4.222	0.012
	3H	−0.728	0.182	8	−3.997	0.017
2H	3H	0.041	0.182	8	0.225	0.996

Note. *p*-value adjusted for comparing a family of 4 estimates.

**Table 13:** ANOVA-electrical conductivity-EFA.

Cases	Sum of Squares	df	Mean Square	F	*p*	η^2^	ω^2^
Time	1.154	3	0.385	269.9	<0.001	0.999	0.9
Residuals	0.000114	8	1.425 × 10^−5^				

Note. Type III Sum of Squares.

**Table 14:** Post Hoc Comparisons–Time–EFA electrical conductivity.

		Mean Difference	SE	df	t	*p*_Tukey_
0H	1H	−0.706	0.00308	8	−229.057	<0.001
	2H	−0.726	0.00308	8	−235.546	<0.001
	3H	−0.716	0.00308	8	−232.301	<0.001
1H	2H	−0.020	0.00308	8	−6.489	0.001
	3H	−0.010	0.00308	8	−3.244	0.047
2H	3H	0.010	0.00308	8	3.244	0.047

**Table 15:** Evidence-based assessment and priorities for subsequent testing.

Sample	Demonstrated Profile	Testing Rationale	Not Demonstrated	Priority Tests
SHA	Strong alkalinity; very high ionic release; K-, Ca-, Mg-, sulphate-, chloride-, and phosphate-bearing constituents	Determine whether processing or controlled incorporation can address ionic-release constraints	Pozzolanic performance; binder and substrate compatibility; durability	Salt release; leaching; efflorescence; reactivity; workability; strength; dimensional stability; durability
EFA	Silico-aluminous and iron-bearing profile; lower conductivity; quartz, hematite, and anhydrite	Investigate behavior as a mineral addition in cementitious systems	Amorphous content; pozzolanic activity; replacement level; fresh and hardened performance	Quantitative mineralogy; loss on ignition; reactivity; water demand; workability; setting; strength; durability

## Data Availability

The original contributions presented in this study are included in the article. Further inquiries can be directed to the corresponding authors.

## Abbreviations

The following abbreviations are used in this manuscript:

SHA	Sunflower Husk Ash
EFA	Electrofilter Ash
SEM	Scanning Electron Microscopy
XRD	X-ray Diffraction
EDS	Energy-Dispersive X-ray Spectroscopy
